# Exploring Airbnb Host Wellbeing and Host-Guest Conflicts in Network Hospitality

**DOI:** 10.3389/fpsyg.2022.805761

**Published:** 2022-02-24

**Authors:** Lucie K. Ozanne, Girish Prayag

**Affiliations:** Business School, Department of Management, Marketing and Entrepreneurship, University of Canterbury, Christchurch, New Zealand

**Keywords:** Airbnb, host well-being, host-guest conflicts, hospitality domains, private-social domain, private-commercial domain

## Abstract

Despite a plethora of studies examining hosting experiences of Airbnb guests, the wellbeing of hosts has received limited attention. Drawing on both top-down and bottom-up theories of wellbeing, we explore the different ways in which Airbnb enhances or diminishes host wellbeing using a multidimensional lens (material, relational and subjective wellbeing). Data is collected from in-depth interviews with twenty-two Airbnb hosts. We also identify tensions and conflicts in the host-guest relationship using the three interactional hospitality domains of commercial, social and private. Through a deductive process, we find that participating in Airbnb both enhances and diminishes the material, relational and subjective aspects of wellbeing for hosts. Inductively, we find that a lack of territorial boundaries in shared accommodation contribute to conflicts that reduce wellbeing. This exemplifies a tension that exists in the private-commercial domain of network hospitality provision. We provide implications for peer-to-peer accommodation providers on developing the managerial skills of hosts, and helping hosts set realistic expectations around hosting to reduce the conflicts and tensions that arise from the intersectionality of the various Airbnb hospitality domains in such a way that the wellbeing of hosts is enhanced.

## Introduction

Airbnb is currently the largest short-term peer-to-peer (P2P) accommodation provider operating in the sharing economy, in terms of listings and bookings (Glusac, 2020). Airbnb enables hosts to rent out their accommodation to guests through the company’s platform. Numerous studies examine the socio-economic benefits of Airbnb from either guest experiences and/or financial benefits to hosts (Xie and Mao, 2017; Li et al., 2019). For instance, Airbnb accommodation offers guests the prospect of staying in a local’s home, a more functional environment (e.g., kitchens, laundry), residential neighborhood advantages, and potential local host interactions (Mody et al., 2017; Guttentag, 2019), among other benefits. An emerging body of literature explores the benefits of participation as Airbnb hosts (Farmaki and Kaniadakis, 2020; Foth et al., 2021), yet hosting experiences remain scantly researched (Prayag and Ozanne, 2018; Canziani and Nemati, 2021; Zhang et al., 2021). In addition, whether Airbnb enhances or diminishes the wellbeing of individuals who participate in the practice needs further research (Buhalis et al., 2020). We expand the literature on Airbnb by identifying additional psychological factors that enhance and diminish host wellbeing (Fischer et al., 2019).

In this study, we conceptualize wellbeing as a multidimensional concept, including material, relational and subjective dimensions (White, 2010). The strength of this approach is that it recognizes that material and objective dimensions are important, but that they are experienced within a relational context where subjective assessments of feeling good about one’s place in the world and one’s role are equally important (Gough and McGregor, 2007; White, 2010; Armitage et al., 2012). In this way, we integrate both top-down and bottom-up theories of wellbeing (Brief et al., 1993).

Airbnb is a form of network hospitality where guests and hosts connect using online networking systems and subsequently interact both online and face-to-face (Molz, 2012). Network hospitality describes how users in our mobile society create relationships with one another by utilizing online networks and the eventual face-to-face meeting for hospitality provision (Wiles and Crawford, 2017). In network hospitality organizations, the focus is on experienced based products and services and great importance is placed on customer reviews through the use of an internet based platform for information exchange and booking, similar to other traditional accommodation providers and other peer-to-peer travel product providers (Wiles and Crawford, 2017). Network hospitality is characterized by five key features, three of which are particularly relevant to our research (Molz, 2014). These include sharing with strangers, intimate but brief encounters, and respatialization of hospitality into private homes. In this study, we explore how these intimate yet fleeting interactions with strangers in the hosts private domain impact host wellbeing.

At the core of this exchange is the provision of hospitality, requiring host-guest relationship management for economic and social benefits (Farmaki et al., 2021). Traditionally, hospitality has been viewed as customer care within an economic exchange framework (Lovelock, 1995), implying a prioritization of customer needs and satisfaction often at the expense of the service provider. Recent studies (Canziani and Nemati, 2021) challenge this notion of hospitality when considered in the context of the sharing economy given that hosts can negotiate and dictate the extent of sharing of their personal space, background and information and time spent interacting with guests. In commercial accommodation and hospitality provision, there are often well established rules and standards on host-guest relationship management, but not necessarily in the network hospitality context. Thus, sharing as a concept moves away from traditional forms of sharing in commercial accommodation and hospitality (Canziani and Nemati, 2021), where relationships with service providers in the sharing economy are more akin to communal relationships (Shuqair et al., 2021b). In communal relationships, individuals not only take care of each other needs but also have a genuine concern for each other’s wellbeing (Aggarwal and Law, 2005). Communal relationships are driven by social aspects while economic exchange relationships are profit-driven (Yang and Aggarwal, 2019), implying that conflicts emerging from communal relationships have to be addressed in ways different to those in exchange relationships (Shuqair et al., 2021b). Accordingly, we use Lashley’s (2000) hospitality domains of private, commercial and social to identify conflicts between hosts and guests that affect wellbeing. Conflicts appear to have become significant Airbnb issues in recent years; however, recent research points to conflicts that emerge among guests rather than between hosts and guests (Ding et al., 2021).

Thus, the research questions of this study are to understand:

RQ1: What aspects of Airbnb hosts’ wellbeing are impacted by managing the network hospitality experience?

RQ2: What types of conflicts emerge from network hospitality hosting experiences and how do these impact on host wellbeing?

### Theories of Wellbeing

Wellbeing is an important concept in many disciplines, including psychology, tourism and more recently consumer research. Given the multiplicity of interest in the topic, there is no unanimous definition of wellbeing (Carlisle et al., 2009). In this research, wellbeing is defined as, “a state of flourishing that involves health, happiness and prosperity” across physical, emotional, social, material, spiritual, environmental, and political dimensions (Mick et al., 2012, p. 6). Psychological theories of wellbeing can be distinguished on the basis of their focus on either bottom-up or top-down effects on happiness (Brief et al., 1993). The two approaches are complementary (Headey and Wearing, 1989; Chen and Yoon, 2019) in the sense that they create a dynamic equilibrium through happiness being derived from a summation of pleasurable moments and experiences (bottom-up theories) but also from individuals’ predispositions to experience and react to events and circumstances in positive and negative ways (top-down theories) (Brief et al., 1993). However, the two approaches to wellbeing have different philosophical roots, having thus different implications for understanding the nature and determinants of wellbeing (Diener, 1984).

Wellbeing has evolved from two main philosophies of hedonic and eudaimonic views, which have been broadened to include other facets (Smith and Diekmann, 2017). The hedonic view focuses on happiness and defines wellbeing in terms of pleasure attainment and pain avoidance, and the eudaimonic view focuses on meaning and self-realization and defines wellbeing in terms of degrees of individual functioning (Ryan and Deci, 2001). More recent conceptualizations of wellbeing call for a holistic view that includes both subjective and objective dimensions capturing material conditions and quality of life (Voukelatou et al., 2021). The objective approach, which is consistent with bottom-up theories (Nakazato et al., 2011), investigates dimensions such as income, job opportunities, assets, and physical health, whereas the subjective approach examines people’s subjective evaluations of their own lives. However, in the tourism and hospitality literature, subjective wellbeing has been prioritized (Uysal et al., 2016; Smith and Diekmann, 2017), with recent studies recognizing different forms of wellbeing (Chassagne and Everingham, 2019) and calling for a more comprehensive assessment of the concept (Holm et al., 2017).

We theorize wellbeing from a social perspective (White, 2010), an approach that transcends the individualistic perspective on how to live well, but also considers relationships with others and the socio-ecological environment (Armitage et al., 2012). This approach identifies three dimensions of wellbeing: material, relational and subjective. Thus, we integrate both top-down and bottom-up theories of wellbeing by recognizing that objective life conditions (Nakazato et al., 2011) interact with individuals predispositions to experience events and circumstances in either positive or negative ways (Diener, 1984) in determining wellbeing. As such, the material dimension includes the physical requirements of life, such as income, wealth, assets, or physical health, and the ecosystem services provided by the physical environment (Armitage et al., 2012). The relational dimension includes social interactions and relationships with others, which pinpoints to wellbeing being a reasonably stable state of an individual consistent with top-down theories. Lastly, the subjective dimension incorporates cultural values, norms, and belief systems, and notions of self, in terms of fears, aspirations, and expressed levels of satisfaction or dissatisfaction (White, 2009). Thus, wellbeing is conceived as arising from the combination of the resources a person is able to command, what needs and goals they are able to meet with those resources, and the meaning that they give to the goals they achieve and the processes in which they engage (McGregor, 2007). This is argued to be as a superior approach to conceptualizing wellbeing as, “wellbeing can be meaningfully understood only when its subjective, objective, and relational components are recognized in an integrated way” and includes an emic account of wellbeing (Agarwala et al., 2014, p. 446). An emphasis on the social aspect of wellbeing derives from the recognition that individual and community needs and satisfaction are embedded and influenced by others, and thus wellbeing is sought collectively and culturally constructed (White, 2010; Armitage et al., 2012; Agarwala et al., 2014). This conceptualization of wellbeing from a social perspective is adopted to understand Airbnb hosts’ wellbeing, thus integrating both top-down and bottom-up theories.

### Airbnb and Wellbeing

Existing research on sharing systems, such as Airbnb, suggest sharing can support individual (Albinsson and Yasanthi Perera, 2012; Ozanne and Ozanne, 2020), family (Ozanne and Ozanne, 2011) and collective or community wellbeing (Albinsson and Yasanthi Perera, 2012; Ozanne and Ozanne, 2016, 2021; Ozanne, 2019; Albinsson et al., 2021). However, there are calls for more research on the nexus of sharing and wellbeing (Eckhardt et al., 2019). Despite the strong evidence of the experiential aspects of tourism and hospitality affecting wellbeing (Uysal et al., 2016; Holm et al., 2017), only a few studies (e.g., Buhalis et al., 2020) have explicitly examined how Airbnb impacts user wellbeing. Airbnb can create conflicts in communities by, for example, increasing crime and disorder in residential neighborhoods (Ke et al., 2021). This impacts social dynamics within a community that may have implications for both short and long-term resident wellbeing. Host-guest conflicts within a shared hospitality environment such as Airbnb (Ding et al., 2021) can reduce the wellbeing of hosts. Likewise, customers deriving dissatisfaction from service attributes that are not considered important by the hosts (Ding et al., 2021) can have implications on not only repeat visitation but also wellbeing of both hosts and guests.

Accommodation provision that generates “homely” feelings can improve traveler wellbeing (Suess et al., 2020). Compared to hotels, Airbnb accommodation can create a home-like experience, and thus is suited to provide a greater sense of wellbeing to travelers (Suess et al., 2020). From the guest perspective, value can be destroyed by unethical actions of Airbnb hosts and poor communication that diminish guest wellbeing (Sthapit and Jiménez-Barreto, 2019; Sthapit and Bjørk, 2021). Airbnb host–guest interactions can contribute to reducing loneliness for both groups thus enhancing their wellbeing (Farmaki and Stergiou, 2019). Buhalis et al. (2020) point to a range of ways that Airbnb contributes to both value co-creation and co-destruction for different stakeholders. Despite this emerging evidence on the impact of Airbnb on wellbeing, the examination of host wellbeing, the focus of this research, remains sparsely researched. A focus on host wellbeing aligns with the communal relationship perspective emerging in the hospitality literature (Shuqair et al., 2021b), where host wellbeing is as important as guest wellbeing.

### Domains of Hospitality and Conflicts

In network hospitality, social interaction and accommodation exchange occurring *via* hospitality services can confer benefits but also generate conflicts in the host-guest relationship. Benefits and conflicts can be understood from Lashley’s (2000), three independent yet overlapping domains of social, private, and commercial hospitality. As Lashley (2000) states, “Put simply each domain represents an aspect of hospitality provision which is both independent and overlapping” (p. 5). The social hospitality domain considers the social settings in which hospitality occurs and the social functions of hospitality (Lashley, 2000; Ikkala and Lampinen, 2015). In this domain, there is an obligation on guests to behave in a respectful manner, doing no harm to hosts or hosts’ possessions, and respecting host rules and their privacy, but significant obligations also fall on hosts (Lashley, 2008). However, from a communal relationship perspective, hospitality provision under this domain would imply that both parties benefit from social exchanges (Shuqair et al., 2021b). Yet the benefits and costs of tourism are borne unevenly by individuals and communities (Lashley, 2008) given the negative impacts of Airbnb (Guttentag, 2015; Prayag and Ozanne, 2018; Hassanli et al., 2019; Buhalis et al., 2020). For instance, social exchanges in the sharing economy can undermine the wellbeing of actors by exacerbating inequity (Hassanli et al., 2019; Davlembayeva et al., 2021; Ram and Tchetchik, 2021).

Whilst there are cultural and social expectations in the provision of hospitality, defining broadly the rules in managing host-guest relationships, network hospitality is experienced predominantly in the private domain (Lashley, 2008). The private hospitality domain, thus, considers issues associated with both the provision of accommodation services in the home as well as the impacts of the host-guest relationship (Lashley, 2000). From this perspective, Airbnb accommodation providers are operating in the private domain with a commercial and/or social focus. The private domain introduces hosts and guests to the rules, rituals, norms and mores, which shape hospitality activities in a social setting (Lashley, 2008). In this way, Airbnb hosts can set limits to the crossovers between the private and social domains within the commercial domain of hospitality.

The commercial domain concerns the provision of hospitality as an economic activity (Lashley, 2000; Ikkala and Lampinen, 2015). It has evolved into well-defined rules, processes and procedures in managing the host-guest relationship (Lovelock, 1995). The rules of engagement between commercial hosts and guests are predefined by the economic nature of the contractual relationship between the two parties, which sets expectations about value and performance. Yet, the standardization and systemizing processes that define commercial accommodation providers such as hotels and restaurants have been criticized for effectively removing the “hospitality” from the transaction (Lashley, 2008). This has also been noted in Airbnb research where commercial hosts tend to operate in a manner similar to hotels (Prayag et al., 2019), thus removing the social experience that guests might be seeking.

Network hospitality that takes place *via* Airbnb can be situated at the intersection of these three domains (Lashley, 2000; Ikkala and Lampinen, 2015) given that: (1) it occurs in the private domain, with a commercial focus and varying degrees of social interactions embedded in the hosts own preferences and customs; (2) has a commercial element as hospitality is monetized; and (3) includes a social aspect as both hosts and guests determine the extent of engagement and interactions online, through the platform, or face-to-face during the stay.

## Materials and Methods

Grounded in an interpretivist paradigm, we conducted in-depth interviews with twenty-two hosts in the Canterbury region of the South Island, which is the second largest region by population in New Zealand. Interviews were conducted between May 2018 and December 2018 before the COVID-19 pandemic. Airbnb listings have grown rapidly in this region from an initial 0.7% of accommodation listings in 2016 to 21.6% of listings in 2018 (Christchurch City Council, 2018). An interpretivist stance has the purpose of understanding the meaning individuals attach to their actions within a particular context, allowing researchers to comprehend the complex process by which individuals construct, negotiate and enact their lived experience (Williams, 2000). In-depth interviews are valuable for providing information on issues that are complex and cannot be directly observed (Malhorta et al., 2017). They provide the scope for probing the context specific meaning of actions and behaviors that uncover how a phenomenon under study is individually and socially constructed (Denzin and Lincoln, 2005). Semi-structured in-depth interviews were chosen as the best method to obtain detailed information and opinions from hosts (Denzin and Lincoln, 2005). We employed a semi-structured interview guide so informants could explore issues that were important to them.

A convenience sampling process was used to identify host participants. We utilized a snowball sampling approach, beginning with the researchers’ contacts, and expanding these to include other Airbnb hosts (Noy, 2008). The following selection criteria were used to identify hosts. First, they must be registered on the Airbnb platform (Farmaki et al., 2021). Second, previous experience with hosting, including both positive and negative experiences, were necessary for inclusion in the sampling process. Third, sample selection considered a variety of hosts (e.g., professional, semi-professional and casual) as suggested by Ikkala and Lampinen (2015). Fourth, to ensure an adequate diversity within the sample, consideration was given to the age, length of experience hosting and host type (Farmaki et al., 2021). The interviews ranged from 1 to 2 h in length, were primarily conducted in the informants’ homes or place of business, and were audiotaped and transcribed. The principle of data saturation was used to determine when data collection should stop, or when no substantive additional idea or theme emerges (Guest et al., 2006). This occurred after twenty-two interviews (see Table 1).

**TABLE 1:** Participants.

Participant	Gender	Age	Years using Airbnb	Education	Type of accommodation	Previous hospitality experience
H1	Female	46	2.5	High school	Entire property	Yes
H2	Female	55	5	University	Entire property	Yes
H3	Female	34	6	Diploma	Entire property	Yes
H4	Female	51	2	High school	Entire property	No
H5	Female	30	2	University	Entire property	No
H6	Male	35	1.5	Diploma	Shared	No
H7	Female	50	3	Diploma	Entire property	No
H8	Male	66	1.5	High school	Shared	No
H9	Female	56	.5	High school	Entire property	No
H10	Female	57	2	High school	Shared	No
H11	Male	66	3.5	Post-graduate	Shared	No
H12	Female	38	1	University	Entire property	Yes
H13	Male	26	1.5	Diploma	Entire property	No
H14	Female	45	.5	University	Entire property	No
H15	Female	59	2.5	University	Shared	No
H16	Female	62	1	High school	Entire property	No
H17	Female	63	3	Diploma	Shared	Yes
H18	Female	63	2	High school	Entire property	Yes
H19	Female	55	1	Certificate	Entire property	No
H20	Female	56	.8	University	Entire property	No
H21	Female	55	2	Post-graduate	Entire property	No
H22	Female	63	1	University	Entire property	No

An important strength of qualitative approaches is that substantive topics can be examined in greater depth. These approaches are also open-ended allowing for potentially new and surprising theoretical insights to emerge (Denzin and Lincoln, 2005), which applies to the inductive approach we use. While there are few specific studies examining the wellbeing enhancing or diminishing aspects of hosting *via* Airbnb, we drew inspiration from studies in different fields (White, 2010; Smith and Reid, 2018) to inform our interview protocol. The interviews started with generic questions about the hosts’ use of Airbnb, type of accommodation provided, frequency of occupancy, among other initial questions. We then asked about the level of interaction with guests, their motivation for hosting, and their best and worst experiences with hosting on Airbnb. Although these initial concepts guided the construction of the interview protocol, emergent concepts evolved within the open-ended structure of the qualitative approach (see Appendix for a sample of the interview questions that were used to prompt the informants).

Our analytical approach fits within a hermeneutical framework (see Thompson, 1997 for a detailed explanation). Data analysis was conducted using thematic analysis. Thematic analysis is the interpretation of data using coding systems to identify patterns and themes, and to discover relationships between them (Braun and Clarke, 2006). This involves the identification of themes through “careful reading and re-reading of the data” (Rice and Ezzy, 1999, p. 258). However, we used a hybrid approach of thematic analysis that incorporates both a data-driven inductive approach (Boyatzis, 1998) and a deductive *a priori* template of codes approach (Crabtree and Miller, 1999). The authors used the three broad types of wellbeing, material, subjective and relational (White, 2010), to deductively code the data.

All authors coded and analyzed the data. The authors used the six-phase approach to thematic analysis (Braun and Clarke, 2006). Although we present this in a linear, step-by-step procedure, the research analysis was an iterative and reflexive process. This process began with one member of the broader research team, who is Ph.D. qualified, transcribing the interviews verbatim, which allowed them to initially familiarize themselves with the data. The authors then read the transcripts multiple times to create initial codes. From the initial list of codes, the authors then looked at developing themes. This involves identifying patterns in discourse and meaning, and then seeing if they fit in an overarching topic or theme. The next step involves reviewing and the potential themes several times. Braun and Clarke (2006) describes this phase as quality checking, and involves comparing the themes to the coded extracts to gauge whether it is representative of the data. The next step involves defining and naming the themes, and finally writing an interpretation of the themes which entails describing, justifying and reasoning to support the theme.

To attain trustworthiness of qualitative research, Lincoln and Guba (1985) suggest four criteria, which include credibility, transferability, dependability, and confirmability. To attain credibility, both authors reviewed the data multiple times and used peer debriefing to discuss the emerging themes (Lincoln and Guba, 1985). There was a 92% agreement on the final themes, which is in an acceptable range (Miles and Huberman, 1994). To ensure transferability, we have provided both demographic and hosting details for each host, and extensive quotes from participants to support the findings. To ensure dependability, we conducted a dependability audit (Lincoln and Guba, 1985) by enlisting an industry expert in the hospitality and tourism field to review our data and themes. To ensure confirmability, both authors kept a reflexive journal to capture fieldnotes and reflections on data collection, tentative interpretations, and plan ongoing data collection. The authors then regularly came together to debrief before agreeing on final themes.

## Results

Table 1 provides a summary of host demographics. Hosts ranged in age from 26 to 66 years old, with the average age being 51 years old. The vast majority of hosts were female, with only four male hosts. The length of time involved in hosting varied from 2 months to over 6 years. Only six of the hosts reported prior experience in the accommodation or hospitality sector.

Next, we discuss our findings. We divide our findings into two sections. First, we describe the wellbeing themes that we deductively identified in the data in terms of material, subjective, and relational wellbeing. We discuss these in terms of how wellbeing is both enhanced and diminished by Airbnb hosting. Although we discuss the enhancing and diminishing aspects separately, we recognize that they can be interrelated (Fischer et al., 2019) or can both enhance and diminish wellbeing (Malazizi et al., 2018). We then discuss the findings that emerged from the inductive approach to thematic analysis which capture the conflicts and tensions that emerge between the three overlapping domains of hospitality and how they impact on host wellbeing. Understanding these conflicts allows us to point to approaches for both hosts and Airbnb to help to resolve them in order to enhance host wellbeing.

### How Airbnb Affects Host Wellbeing

#### Enhancing Material Wellbeing

**FIGURE 1:**
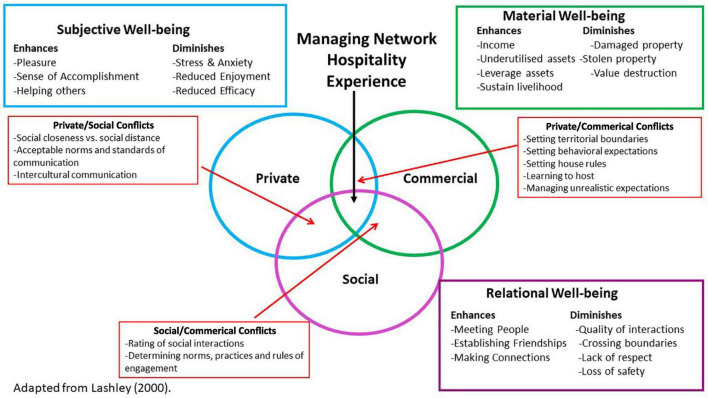
Types of host well-being and host-guest conflicts.

The material aspects of wellbeing include consumption levels, livelihoods, and wealth (White, 2010). In the commercial hospitality domain, our results reveal that Airbnb provides hosts the opportunity to enhance their material wellbeing (see Figure 1) through providing a supplementary income in the form of short-term rent for their property (Fang et al., 2016; Lampinen and Cheshire, 2016), which is found to be a primary reason for hosting (Farmaki and Kaniadakis, 2020). However, some literature points to the fact that financial aspects can be both a gain and a pain for hosts (Fischer et al., 2019). For instance, hosts discuss a key motivation for using Airbnb is the extra income, which allows them to pay their mortgage, rates and other personal expenses, or to undertake property renovations or maintenance.

The primary factor was that the apartment was sitting empty and we thought ‘ok, we could rent it out a couple nights a week, which would help pay the bills’. (H4)

It’s easy because it’s easy money! (H8)

Get some money in and pay our mortgage! (H20)

Hosts also prefer Airbnb rather than renting their property to long-term tenants, as they felt short-term rental would generate more income, as discussed below:

And then from there it was kind of ‘rent is only so much but people are willing to pay a lot more for a short-time accommodation’. I only pay NZ$100 a week for a room but people who visit us…. a hotel or a motel are paying NZ$100 a night. So we can actually cover actually a lot more, it helps to financially sustain, yeah! (H13).

A secondary but still important material benefit was that Airbnb allows hosts to rent out underutilized assets, as the quote below illustrates. Thus, rather than having unrented properties, hosts were able to enhance their material wellbeing by generating income from these assets. This material benefit has been previously argued in the literature (Oskam and Boswijk, 2016; Petruzzi et al., 2021), with some empirical evidence supporting this benefit (Ikkala and Lampinen, 2015; Buhalis et al., 2020).

I was just sort of to give something a go! If you don’t give anything a go, and the rooms were just sitting empty… so we thought ‘I could do that, it’s so easy!’ (H8)

Other respondents purchase properties with the explicit goal to rent them *via* Airbnb, as the quotes below illustrate. They view the property as an investment and Airbnb as a tool that will enable them to gain both short and long term returns on investment (Farmaki and Kaniadakis, 2020), enhancing their material wellbeing. This was a particularly important material benefit for those nearing retirement.

…the returns are quite good, so we thought we’ll give it a go. So yeah, it was just purely investment…(H9)

So it’s a short-term thing. We have put some value into it, we are providing a good service in the meantime and hopefully, there will be some capital gain at the other end. But in the meantime it’s as good of an investment as putting money in the bank. (H21)

Because we were due to retire in a couple of years so it’s a good way of hopefully growing what money we’ve got in a property. (H22)

Finally, some respondents use Airbnb as either a full or part-time job. Thus, managing an Airbnb provides them a livelihood, or the opportunity to become a microentrepreneur (Zhang et al., 2019). As one respondent describes, “As an income, and just to have a little business, yeah!” (H17). This reflects planning by respondents who believe that Airbnb will provide greater flexibility than a full-time job, and thus will enable them to undertake a lifestyle they aspire to. For instance, one participant describes being able to work less than her previous full-time job, while still maintaining her income and pursuing activities she enjoys:

No. It is literally a job for me! …Lifestyle, yeah, I think lifestyle is the biggest one… And so he said to me (her husband) ‘I worked out that if we run the unit as accommodation, and you did that as your job, for 25 h a week you could earn what you are earning now’. So I was quite happy to swap over. (H19)

Other respondents use Airbnb more reactively. For instance, one respondent describes using Airbnb as a part-time job when they were made redundant. Thus, Airbnb provides a critical method to support this individual financially and support their material wellbeing.

I got made redundant. I did get a part-time job, but I did need to supplement my income, so a friend said to me…And she said to me that she had a friend who had just started doing Airbnb, and was making heaps of money, that I should try it…. And as many people like myself post-quake who are older, we actually need this income, you know. To actually survive and keep our homes, to keep afloat. (H15)

### Diminishing Material Wellbeing

In the commercial hospitality domain, while Airbnb provides a number of financial benefits that enhance material wellbeing, it can also diminish host wellbeing. For instance, many hosts point to incidents where contents in the property were damaged, or the property had been left in an untidy state (Buhalis et al., 2020). Most hosts describe these incidents as being more a nuisance than a serious concern, as illustrated by the first quote. However, they do involve effort on the part of the host to return the property back to a presentable state for the next guest, as illustrated by the second quote.

I’ve had a couple of things broken but that’s you know? …and flooding the bathroom, rather than telling me that it’s wet so that I can go and mop up but it’s nothing too major…(H10)

Guests don’t have to make beds but things thrown everywhere, food left in the oven. Messy toilets, condoms… you know… the whole nine yards as far as cleaning up that mess in a short amount of time, is very difficult. (H1)

However, some hosts discuss more serious incidents that involve much greater property damage and thus substantially reduce material wellbeing for the host. For instance, hosts describe having items stolen, guests doing substantial property damage, and in one case the property being burnt to the ground, as described below.

They left the fire door open and went upstairs, and they say that their kids opened the door, but we don’t necessarily think that we think that they left the door open and that they went upstairs. And a log rolled out, and the whole house burnt down. (H5)

In addition, these incidents point to a considerable amount of value destruction of host assets, and substantial effort on their part to return the asset back to its former state. In addition, some hosts describe that when these events occur, Airbnb is not supportive of them in trying to restore that value, as is described by host 21 below. Host 19 goes further and describes having to take legal action to recover compensation for the property damage.

I had to replace my benchtop the first time, I have had to do it twice, is because an Airbnb guest put a hot pot down on it and burnt right through it and when I contacted Airbnb they just didn’t want to know. They were supposed to provide all this support for owners and things, and they were just like oh no c’est la vie. I wasn’t very happy about that. (H21)

I had to take her to court in the end to get the money out of her but I did. So we had it fixed the next day (the hole in the wall). (H19)

### Enhancing Subjective Wellbeing

The subjective dimension of wellbeing includes people’s perceptions of their material and social positions as well as their feelings, values and beliefs (White, 2010). The private domain of hosting guests *via* Airbnb enables hosts to enhance their subjective wellbeing in various ways (see Figure 1). For example, hosts describe feelings of pleasure when they receive a positive review or an expression of gratitude from a guest (Ikkala and Lampinen, 2015; Malazizi et al., 2018), as described below:

And I had well even a review that I got today from a guy who was just there one night ‘it was absolutely lovely’ and loves it and he wants to come back…I think that the best ones are when people either go out of their way to not only leave you a review, but they will message you and say, you know, this has been really good, thanks for going out of your way. (P4)

Previous research has found that hedonic benefits positively influence perceptions of value of Airbnb guests (Stollery and Jun, 2017), while our results show that these pleasurable feelings are also important to hosts enhancing their subjective wellbeing.

Hosts also describe a sense of personal accomplishment as guests show appreciation for various aspects of their property (Zhang et al., 2019), providing eudaimonic feelings for hosts (Ryan and Deci, 2001). This might include appreciation for their garden, house, or the manner in which they run their Airbnb property. In the first quote, the host describes the sense of accomplishment they feel based on a guest’s praise of their property. In the second quote, the host explains the sense of achievement they feel when guests recognize the environmental values they use to guide the management of the hospitality experience.

He sent me a text to say what an amazing facility it was, everything was so perfect and he just couldn’t believe it and it was like ‘oh my god, I just love this guy’. And I just thought that from Auckland their expectations are very high, but yeah, I was blown away so that was really, really good. (H9)

…the whole purpose of our business is to inspire people to live more eco-friendly…Or it’s people in appreciation of what we’ve done, which is really nice, they’ll be like ‘wow, your kitchen’s amazing’ and ‘oh, I love this and I love that’, it’s quite nice to have interactions like that. (H3)

Finally, hosting through Airbnb provides hosts the ability to help guests navigate their stay, which many hosts enjoy, expanding our understanding of the psychological factors that impact hosting (Fischer et al., 2019). This might include the ability to help them navigate the city, find a restaurant or other amenities, or find other ways to enhance their stay, as the quotes below illustrate.

We’ve had a few families like that and that makes me feel like… you haven’t really done much but that maybe helped to make their trip. And really all we are doing here is making it easy for people to either be in the city or go somewhere else. (H7)

When I go somewhere, I wish I had a friend there and that’s something I feel like we are as a whole team, people come down here and go ‘where should I go to eat?’ you know? ‘What’s happening in the city?’ we are so well connected that it’s usually really helpful and people really appreciate our help…(H13)

Hosts also appreciate being able to provide an enjoyable hospitality experience for guests or to offer their hospitality to guests (Ikkala and Lampinen, 2015). As the two quotes below illustrate:

It was really nice to know that they were enjoying it. And they just…they’ve loved the property and that was really lovely! Just knowing you are providing a nice service for them that was a nice feeling I thought. (H14)

Comfortable, and safe are not quite the word, like giving them permission to actually make my house their house while they’re there. And so I tried to put everything in place to make people feel like it’s their home as well as my home while they’re there. (H15)

In essence, hosts serve as brokers when they manage the Airbnb experience for guests helping to frame what the experience is for the guest (Angelini, 2020). In this role, they make it easy for others to complete their trip and understand the space and place by guiding the guest to specific products and places and consumption experiences. From the host’s perspective, serving in this role is pleasurable and enhances their subjective wellbeing.

### Diminishing Subjective Wellbeing

Despite the private domain of hosting guests enabling hosts to enhance their subjective wellbeing, managing the Airbnb accommodation experience can also diminish host subjective wellbeing. For instance, hosts describe the stress and anxiety they experience, when despite their efforts to accurately describe their property or evaluate the suitability of guests, incidents occur when the guest is not happy causing stress for the host.

Or when they’re disappointed about the property. We have a property that has no TVs, and it says it in the listing, and people arrive and they’re furious that there’s no TV because they’ve got children who have to watch TV…So it is exhausting sometimes when you think that you have vetted somebody correctly, you’ve gained and gathered all the information you feel that you need to ensure that this is going to be the right guest for the right property. And when it’s not, it’s disappointing… (H5)

Another cause of stress for hosts that reduces their subjective wellbeing is when guests leave bad reviews, which indicates that reviews can either enhance or diminish wellbeing (Fischer et al., 2019). While some hosts feel that negative reviews are helpful, most would rather not receive negative reviews. In addition, many of the hosts feel the negative reviews are not fair, as the guest has not thoroughly read the property description, as described in the quote below:

Yeah, so here is a great example: they’ve given, and this is for my one-bedroom apartment they’ve paid NZ$96 per night, they’ve given me three stars, they say ‘the location was sketchy and not a short walk from town as it was located in a very industrial area’. So that’s a lack of research on her behalf, it’s not advertised as being a short walk from town, so her expectations were certainly her own, yep! So yeah, I don’t know, you just can’t keep everyone happy, yeah! (H12)

Individuals enter into social interactions hoping for social rewards, including approval or respect (Blau, 1964), when they receive negative social rewards, this can be disappointing for hosts.

Another way that host subjective wellbeing is diminished in the private domain of hosting is through the practices of Airbnb. Many of the hosts in our study describe regular campaigns by Airbnb to encourage them to reduce their pricing or offer additional services. For some of these hosts, this practice has changed the experience of hosting and reduced their sense of enjoyment, as this host describes:

But we are now getting sick of Airbnb, I would say every ten days sending stuff saying that we would have more guests if we reduce our price. Have we thought about offering extras like tours of the city?… But now with Airbnb on your neck all the time, reducing prices, offering more, it has changed the nature of it really. (H11)

Others describe that Airbnb practices, have reduced their sense of self-efficacy with hosting. For instance, the host below initially followed Airbnb’s advice to set a low price for their property. However, they always felt that this price did not accurately represent the value of their property. They subsequently raised their price, but Airbnb continual efforts to get them to reduce the price, have contributed to a lack of confidence in their hosting abilities.

Then we went to another pricing after that NZ$35 and then we went up to NZ$60. So there you go, that’s what we were and now what we’ve done over the winter, because Airbnb came back and said that we had to ‘drop it’, out price for the winter months and we both decided that it’s not important if we get people in or not. (H16)

Like the hosts in the study by Farmaki and Kaniadakis (2020), some of the hosts in our study feel disempowered against the positional authority of Airbnb who they feel dictate changes regarding their hosting practice, which leads some to stop using Airbnb, as described below:

As well as that they’ve (Airbnb) now got these new criteria so you have to have five good families stay for them to have them advertise you as family friendly. So you have to have five good family reviews. So they have changed the way that they do that. So most of us are dropping off the Airbnb site because we just haven’t got the number of reviews because we use a number of different sites. (H21)

### Enhancing Relational Wellbeing

The relational aspects of wellbeing include social relations, attitudes to life, and personal relationships (White, 2010). In the social hospitality domain, Airbnb provides hosts the opportunity to enhance their relational wellbeing in a number of ways despite guests being strangers who enter the space for brief encounters (Molz, 2014; see Figure 1). Specifically, Airbnb provides an opportunity to improve the hosts’ social connectedness with other people (Ikkala and Lampinen, 2015; Malazizi et al., 2018). For instance, through having people share space in their home, Airbnb provides hosts the opportunity to meet and interact with people as one host describes their motivation for hosting, “…so then having that interaction with the guests” (H5). These interactions may serve as a method of reducing loneliness for hosts, and take the form of either temporary interactions or more meaningful interactions that extend beyond the Airbnb stay (Farmaki and Stergiou, 2019). For instance, for many hosts in our study, these interactions go beyond just “meeting people and having a chat” to developing personal relationships with those guests who they share similar interests or with those from other cultures, as these quotes capture:

The best would be we had quite a few couples about our own age who were really interesting and a couple who we had an evening meal together. (H11)

I suppose that because I had the opportunity to live and work overseas for 10 years, it’s kind of like I travel vicariously through the guests that come. And a lot of times they come from places that I used to live in… I just enjoy meeting people from those countries and talking about it. But also sharing what it’s like to live in Christchurch. (H15)

…it’s really cool having cultural experiences. We had a group coming from Argentina and they stayed here for ten nights and they were here for the golden oldies rugby and they were really sweet you know? That’s just such a lovely culture, they are so welcoming and inviting. (H3)

As these quotes illustrate, these social interactions provide the opportunity to build relationships with others through sharing a meal or sharing personal experiences. They also enable guests to co-create value with hosts. While the literature identifies that hosts serve as a key resource that form the basis of collaborative value co-creation efforts in Airbnb settings for guests, our findings reveal that guests can play the same role for hosts (Johnson and Neuhofer, 2017). As guests share cultural knowledge about their home, they create value for hosts that enhances their wellbeing. As Dolnicar and Talebi (2020) explain, hosting is seen by some as an experience similar to travel itself with the host becoming a visitor to the guest’s country of origin without leaving their home.

Some hosts describe how these relations go beyond the guest’s visit and develop into longer term friendships that enhance their relational wellbeing (Farmaki and Stergiou, 2019).

We’ve got someone from France who actually has become a really good friend. We’ve invited him over and back to come and spend Christmas with us. (H6)

It is an opportunity to form friendships or relationships with really interesting people. And I don’t know if it’s unexpected because that’s really the reason and it continues to be the positive side. (H11)

In addition, hosts perceive that they may be able to activate these personal connections in the future if they choose to travel or reach out to these contacts.

Well, I thought sometimes it would be quite nice to have some connections if I want to travel myself. (H10)

And we made all of these connections and stuff. Yeah I would say the highlight is that a lot of the people I’m still in touch with. We are friends on Facebook… (H13)

Although the literature shows that financial motivations prevail over social ones (Karlsson and Dolnicar, 2016; Mittendorf, 2018), social motivations are still important for many hosts as our findings support (Moon et al., 2019). With host-guest interactions contributing to the perceived authenticity of the experience (Guttentag et al., 2018), it has been argued that social relationships and a sense of community are sought after by Airbnb guests unlike those staying in traditional hotels (Tussyadiah and Zach, 2017), they are also important for our hosts.

### Diminishing Relational Wellbeing

Although most hosts really appreciate the opportunity to interact with guests and develop personal relations in the social domain of hosting, there are also elements within this domain that diminish host wellbeing. For instance, some hosts find that the nature or quality of the personal interactions are poor, which can be time consuming and frustrating for hosts, as this host describes:

He wouldn’t interact too much with me. It was just something about him. I don’t know what exactly it was, but yeah… I think it was just that arrogance with trying to talk to him. (H10)

For most hosts, the poor quality of these interactions is just tedious in nature and does not cause major concerns. However, for other hosts, guests sometimes cross physical and personal boundaries that indicate a lack of respect for the host and their possessions. As these hosts describe, this happens when guests come into areas of the home or use things that they are not allowed to, showing a lack of respect for the hosts and their property:

But they’ll just come in… we came back an hour and a half back later and by then they had been through the house, they put stuff in the fridge, taken stuff upstairs… so they thought… I think for them it was “we’ve got a room like we would have in a hostel, we’ve got it to ourselves and the rest of the house must be for us… But it is clearly stated and it’s probably once every couple of months that someone comes expecting that they can use the kitchen. (H11)

As Ravenelle (2020) explains, hosts believe that guests should remain within the boundary of their stay and prefer guests who respect the hosts’ rules. When guests violate these rules, this can diminish relational wellbeing for the host.

For a few hosts, guests behave in such a manner where they feel their privacy is breached, which reduces their perceptions of safety, negatively impacting their relational wellbeing (White, 2010). In the quotes below, female hosts describe how male guests venture into areas of the property they are not allowed, or behave in a manner they are uncomfortable with.

And I’ve had had a couple of people where I’ve said ‘don’t come any further, I don’t want you in here’. A couple of them, they didn’t know, I think they were male too. (H10)

I wouldn’t have been very comfortable dealing with them on my own. Like I went in to set up the TV, because we’ve put the rugby on for them in our living room and there were some pretty lewd comments because they were drinking and stuff. (H3)

While previous research has found that safety and security risk reduce the guests’ satisfaction and intention to continue using Airbnb (Malazizi et al., 2018), we find that guest behavior can also create negative social interactions that reduce the host’s relational wellbeing.

### Hospitality Domains, Host-Guest Conflicts and Impact on Wellbeing

In each hospitality domain, we find that hosting through Airbnb can either enhance or diminish host wellbeing, despite hosts offering entire properties for rental rather than just shared facilities. The very nature of network hospitality provision entails the commercial and social domains intruding on the private domain, creating interesting host-guest dynamics (Lashley, 2000; Molz, 2014; Wang and Li, 2022). Through our inductive thematic analysis, we find that conflicts and tensions arise from the overlapping of the various hospitality domains that need to be resolved and managed, which we discuss next (see Figure 1).

### Private-Social Conflicts

When hosts rent space in their homes, they bring the social domain into their private domain (Molz, 2014) that can generate conflicts that need to be resolved. By renting the entire home or opening the home to strangers through shared facilities, hosts (personal peer vs. commercial peer providers—Shuqair et al., 2021a) provide hospitableness through, for example, sharing meals, providing advice and interacting with guests. Social closeness (or social distance) with hosts affects the customer experience (So et al., 2019). However, as one host describes, “we try to make them [guests] feel at home but you don’t actually often get to know them that well because you can’t really delve into what they are doing” (H11).

For some hosts, what constitutes acceptable levels of engagement, interactions and involvement with guests can be problematic leading to diminished wellbeing. As one host said:

You just felt like that this was somebody who needed to talk non-stop for hours and need to be heard. And then he came and stayed again and we went ‘oh no’… we had a lovely experience but it was taxing. (H3)

The quote above also illustrates that acceptable norms and standards of communication from the host’s perspective were transgressed by the guest. Due to “hospitableness” in the host-guest relationship, the host could not stop the conversation with the guest, thus, having a negative impact on the host’s subjective wellbeing.

If hosts are able to negotiate the degree of social closeness desired by both parties, this can enhance the hosting experience and positively contribute to their general welfare as indicated in the quote below:

…it’s a two-way thing. I mean, they give and we give too you see. And that’s a mutual sort of relationship, isn’t it? When people interact like that and we are all in it and that’s good. That’s happens a lot! And there are the odd person that you don’t feel very comfortable with…and they’re just a bit picky…(H17)

The quotes above illustrate both benefits and conflicts emerging from the intersection of the private-social domain of hospitality. Both hosts and guests ascribe different value to social interactions and the meaning of hospitality in the host-guest relationship. This is because there is no set acceptable norm and standard of hospitality provision that is specified by Airbnb unlike in commercial accommodation provision (e.g., hotels). Thus, hosts have to determine and set their own rules of engagement and interactions with guests.

In addition, hosts have limited ways for understanding the desired level of social interaction guests expect. In particular, inter-cultural communication can be problematic, as shown in the quote below, which affect both guest and host experiences, resulting in reduced material wellbeing for the host:

But I had some people from China… They arrive here, and I said ‘is everything ok?’ they said ‘yes, everything is fine’. So I get a text saying that they are leaving the property tonight. The house wasn’t what they had expected. So I rung up the big bosses from Airbnb and they wanted me to refund the money, ‘well I can’t do that’…I should have taken $NZ30 for the house or whatever. Because it takes quite a while to get the rooms and everything all ready for them. And I’ve bought food and stuff in for them. And of course, they hadn’t used Airbnb before either. They were first-timers. (H8)

### Private-Commercial Conflicts

Wang and Li (2022) suggest that home territoriality is important to guests (i.e., accessibility, house rules, signs of ownership, and host intrusion into guest space) and can diminish their sense of satisfaction with the Airbnb experience. From the standpoint of host wellbeing, it is critical for hosts to set territorial boundaries around the extent of intrusion of the commercial domain into the private domain. However, our results show that the extent to which hosts are able to set clear boundaries in the private domain and manage these for guests’ access to shared facilities and amenities as well as the extent to which guests respect these boundaries can be problematic. Material wellbeing is diminished when hosts fail to properly set rules or create boundaries for guests. In one instance, participant 11 mentions:

We had couple of Asian girls who obviously stood on the toilet seat and broke the toilet seat. We had a guest who had obviously eaten in bed and you get sheets with stains that wouldn’t come out. (H11)

In essence, what constitutes “shared” space is not always understood by guests due to their lack of familiarity with Airbnb or the expectations of both hosts and guests in such settings. As one host mentioned, the guest ends up encroaching on the private space or territory of the host, as illustrated below:

I had two ladies staying here for 2 days, and they had two dogs with them. And the ad says ‘no dogs allowed’ but they kept them in cages because they were show dogs. But it was wet and it was horrible and they had to wash the dogs’ towels and stuff. And I didn’t want that did I? But the lady told me that she hadn’t done it, but I knew jolly well she had washed damned stinking old dog towels in the washing machine… and then she put all the laundry all around our fire place there and hang it all out and she just took over the place really. (H17)

These behaviors, while tolerated in the commercial accommodation context (e.g., hotel) as they would be attributed to normal wear and tear in service provision, in the sharing economy context, such behaviors are perceived by hosts as being unacceptable in their private domain. The transition to using private property in the commercial domain blurs the boundary of who is the custodian of the amenities and facilities, leading to diminished wellbeing of the hosts, in many instances.

In addition, the nexus of private vs. commercial accommodation domains can also be a source of confusion for the guest, which contributes to negative experiences for the host. Behaviors commonly practiced in hotels by guests are replicated in the Airbnb accommodation, which are subsequently deemed unacceptable by hosts as illustrated in the quotes below (e.g., dropping litter, cigarette butts). Yet, the private-commercial hospitality nexus entails setting clear expectations for guests (e.g., number of guests), which in this case was not clearly communicated to the hosts. Lack of clarity in house rules can also contribute to the “home” being used as a hotel by guests.

Yeah and they’ll just drop litter around and treated like… you can tell that they’ve treated it like a more like a hotel. Or they might be new to it and not realise that they got to be actually a bit more respectful. (H7)

…and the worst was a young group of people, which was meant to be four people coming for a weekend, and it ended up being a whole lot more people. My house-it wasn’t trashed- but then there was soft drinks spilt on the carpet, all the bed were used actually and not paid for. Cigarette butts all around in my pot plants and when they left, they left the house wide open. (H14)

Transitioning from hosting in the private to the commercial domain can be a steep learning curve for hosts. This can induce uncomfortable feelings, as hosts have to learn what commercial hosting entails and the boundaries that must be set with guests. As one host said,

That’s a really fine line, really. And it took me a while to learn that. So to begin with I was probably a bit overpowering. You know? ‘can I do anything for you?’. You do this and that and [name of her husband] would be saying to me ‘let go [name of H16]. And then I let go and then it would probably not be enough. So it took me a while, probably it took me a month worth of guest to work out how much to interact with people. (H16)

Unrealistic expectations of hosts as they transition from private to commercial hospitality can also be seen in the experiences of some hosts. These unrealistic expectations were sometimes related to amount of money they could make from Airbnb, how much work they will have to do for managing the property and also who they will meet through hosting experiences. As illustrated below, the host was expecting to have a mix of guests over time but she was disappointed as most of her guests were young people. Relational wellbeing can be negatively impacted until the host find ways to reset their hosting expectations.

…but instead, we had all these young people coming through! Well, of course, because it was cheaper than a tent site…and I went and said to him [name of her husband], you know ‘I don’t want to see any more young people anymore’, you know? ‘I had enough of this’… I just wanted some older people to have conversations with and everything you know? (H16)

### Social-Commercial Conflicts

The lack of a mutually beneficial relationship between hosts and guests and a host lack of concern for his or her guest can contribute to perceptions of negative experiences from the guest perspective (Sthapit and Bjørk, 2019). However, it should also be noted that conflicts are at play in the post-hosting experience in relation to guest online rating of the host and the property. Conflicts in the social-commercial domain can reduce subjective wellbeing of hosts when guests are perceived as untruthful in their rating of social interactions with hosts and when the online comments about the property are perceived to be unfair. As one host stated:

I think my worst hosting experiences have been in retrospect those two people who ended up being critical afterwards about the fact that I had misrepresented myself by saying it had been an entire house when I lived on site. (H15)

In the social-commercial domain, lack of hosting experience can often undermine how hosts perceive the norms, practices and rules of engagement used by guests to evaluate the Airbnb experience. Insufficient clarity on how guests determine and rate various aspects of the experience can diminish the wellbeing of hosts. As illustrated in the quote below, the guest is commenting on the experience in a negative way on factors that are outside the control of the host. Thus, the norms and standards being used by the guest to judge the experience are perceived as erroneous by the host. Despite the host being open to critical feedback that can enhance to the hosting experience, in this case he felt hopeless.

And it’s good to have that feedback because that’s’ how you improve but yeah. Sometimes you feel a bit hopeless when there are things like the dark scary walk and I’m like ‘this isn’t an ideal ‘location’. And I wouldn’t say that it is negative feedback it’s more when it’s critical feedback and that’s good. That’s actually how you grow and how you improve, yeah…(P13)

In another instance, the online rating did not match the written feedback as illustrated in the quote below, which led to an unhappy host. This illustrates the discordance experienced by hosts when guests do not adhere to expected rules of engagement in the sharing economy. There should be a congruence between the experience provided by the hosts and the online rating they receive but the reality can be different.

And then this one, this one gave me three stars but their public feedback ‘was nicely furnished and finished apartment-I’m not quite sure what that means- and they didn’t give me a five start for anything but there was no feedback on why. So yeah, I don’t know, you just can’t keep everyone happy! (P12)

## Discussion and Implications

In this study, we examine whether managing the network hospitality experience across the private, social and commercial hospitality domains enhances or diminishes Airbnb host wellbeing. In this way, we contribute to the nascent literature examining the impact of different forms of sharing on wellbeing (e.g., Buhalis et al., 2020; Ozanne and Ozanne, 2020). We find that providing hospitality to strangers (Molz, 2014) in the private domain can both enhance and diminish host wellbeing across the material, relational and subjective dimensions. In this way, the findings provide support for bottom-up theories (Diener, 1984) of host wellbeing in that objective life conditions related to material wealth (e.g., how guests utilize the accommodation property) affect host wellbeing. Likewise, hosts wellbeing are either enhanced or diminished by, for example, guest comments on the property and hosting experience, providing some support to positive and negative effects on host happiness, aligning with top-down theories of wellbeing (Brief et al., 1993; Chen and Yoon, 2019).

In regard to material wellbeing, hosting provides hosts an income, an opportunity to utilize assets, an investment opportunity, and method to sustain their livelihood. However, theft and damage to property diminishes host wellbeing through value destruction of these assets. In regard to subjective wellbeing, despite the usually brief encounters with guests, hosts describe pleasurable feelings and a sense of accomplishment from hosting (Fischer et al., 2019). Hosts also enjoy helping guests to navigate their Airbnb stay. In this way, the experiences provided through Airbnb hosting confirm several characteristics of network hospitality such as sharing with strangers and intimate but brief encounters between hosts and guests that are emotionally charged having an impact (positive or negative) on wellbeing. We find that subjective wellbeing can be enhanced or diminished by guest reviews of the accommodation experience. Host subjective wellbeing can also be reduced for through the actions of Airbnb that diminish the enjoyment of hosting or undermine their feelings of self-efficacy, and some stop hosting. Our findings on the subjective aspects of hosting, thus, expand our understanding of the psychological factors that enhance and diminish host wellbeing (Fischer et al., 2019).

In regard to relational wellbeing, hosting enables hosts to meet interesting people and establish friendships. Yet relational wellbeing can be diminished when the quality of social interactions is poor, and when guests traverse boundaries which indicates a lack of respect for the host or their possessions. The different facets of wellbeing identified in this study, can be related to the traditional notions of hedonic and eudaimonic wellbeing that underpin the majority of studies on psychological wellbeing (Ryan and Deci, 2001). For example, hosts achieve hedonic wellbeing through their participation in network hospitality, offering their home and facets of their lives for the guest to understand and appreciate, thus epitomizing the hospitality to strangers aspect of network hospitality. Through social interactions with guests, hosts can also achieve eudaimonic wellbeing in that they can find a renewed sense of purpose in life. However, both hedonic and eudaimonic wellbeing can be diminished through the hosting experience when hosts are unable to navigate the conflicts that are created by bringing strangers into their home.

The diminished wellbeing among hosts seems to result from the conflicts and tensions that arise from providing network hospitality. This implies that the intersections of the private, social and commercial domains of hospitality generate challenges for the hosts in understanding and managing the host-guest relationship. A fundamental premise of the sharing economy is that social benefits accrue to the participating actors in the host-guest relationship (Farmaki et al., 2021). While we find that social interactions can contribute to both enhancing and diminishing the wellbeing of hosts, the essence of communal relationships, which is based on the wellbeing of both hosts and guests (Shuqair et al., 2021b), is lacking. However, hosts are not always able to determine the extent of social closeness with guests, as they are essentially strangers who they have brief encounters with, and guests can have a role in setting boundaries for social interactions. Thus, in the private-social domain of hospitality both hosts and guests affect the level and extent of social interactions which can diminish hosts’ wellbeing. Yet, genuine concern for the wellbeing of both actors (hosts and guests) which underpins communal relationships (Aggarwal and Law, 2005), ends up being one-sided, where hosts do their best for guests, but the latter shows little concern for hosts’ wellbeing. In fact, guests tend to adopt and replicate behaviors that are focused on their own hedonic wellbeing, as has been the case in commercial accommodation provision, rather than those based on the sharing concept (Shuqair et al., 2021b), which could have improved both hedonic and eudaimonic wellbeing of hosts. In this way, the host-guest relationship reflects more of an economic exchange (Lovelock, 1995) where social aspects are important (Yang and Aggarwal, 2019) but do not necessarily translate into wellbeing for all actors involved. Thus, network hospitality does not always generate positive health related outcomes for hosts.

However, similar to Canziani and Nemati (2021) we found that the economic exchange between hosts and guests is not purely transactional given that hosts can dictate the extent to which they share personal space and information with guests. In network hospitality provision, the extent of sharing can be managed and remains the domain of the hosts. However, hosts are not always capable of managing the sharing aspects of hospitality provision effectively. As our findings indicate that host wellbeing is impacted when the social and commercial domains are not managed effectively leading to a perceived intrusion by the guest on host private domain of hosting. This occurs when rules of engagement, territorial boundaries and communication are not clear between hosts and guest. Thus, despite the host demarcating areas of home as either private or shared, guests do not always respect such boundaries. Thus, conflicts emanate from unsuccessful respatialization of hospitality into private homes by hosts. However, hosts can mitigate the negative impacts on their wellbeing when they find ways to manage the conflicts that emerge between the interrelated domains of private, social and commercial domains of network hospitality provision.

While conflicts in the host-guest relationship is not uncommon in network hospitality provision such as Airbnb (Sthapit and Jiménez-Barreto, 2019; Shuqair et al., 2021a), the extent of social distance or closeness (So et al., 2019) that occurs in the private-social domain of hospitality provision can both enhance and diminish host wellbeing. As there are insufficient guidelines provided by Airbnb around the extent of hosts and guests interactions online and face-to-face, it becomes the domain of the host to determine the nature and level of interactions with guests that will fulfill the latter’s expectations. As shown by our findings, this can become a potential source of conflict. Yet, in commercial accommodation provision, this has been a longstanding issue in customer experience management (So et al., 2019), and as noted in previous Airbnb studies (Ikkala and Lampinen, 2015), the significant social obligations seem to have fallen exclusively upon the host rather than giving guests the responsibility to manage social interactions as well. The rules, rituals, and norms, which shape hospitality activities in a social setting (Lashley, 2008), should be explicitly stated for guests staying in Airbnb accommodation as the lack of these diminishes wellbeing for both actors.

While commercial hospitality provision has been criticized for removing the “hospitality” in the host-guest exchange due to standardization and systemization of service delivery (Lashley, 2008), the lack of these in Airbnb provision has become a source of conflict. In the private-commercial domain, the lack of territorial rules and boundaries negatively affect the hosting experience (Wang and Li, 2022). Thus, in the provision of hospitality, Airbnb hosts can experience diminished wellbeing when private space of the host is violated by guests. This is because of a lack of standardization in facilities and amenities provided through Airbnb. Thus, both hosts and guests do not have a frame of reference to work with in delivering the hospitality experience. The lack of hosting experience can also negatively impact the hospitality provision.

In the social-commercial hospitality domain, the host-guest relationship is not always mutually beneficial as suggested in previous studies (Sthapit and Bjørk, 2019) and echoed in our findings. The conflicts that arise in this domain pinpoint to a lack of trust between hosts and guests but also between hosts and Airbnb as a company. The strong focus on guest experience driven by Airbnb comes often at the expense of host wellbeing, particularly, in the domain of post-experience host rating. As there are no checklist that guides the guest in rating the host accommodation and hospitality provision, there are discrepancies between actual experience provided by the host and guest rating of the host. This asynchronous rating of service provision has long been the domain of service quality provision in commercial accommodation (Lovelock, 1995), where standards and procedures are formalized to manage guest expectations. As these are lacking in network hospitality provision, the social-commercial domain interactions become a significant contributor to host diminished wellbeing. Thus, host wellbeing is contingent on their managerial skills, sense of self-efficacy in managing relations with guests and Airbnb, and ability to manage their expectations around hosting. Our findings support previous research that implicate factors impinging on the correlates of host wellbeing including their managerial skills and sense of competence in hosting (Buhalis et al., 2020).

Airbnb can enhance host wellbeing through a variety of approaches. First, our hosts had limited prior experience in hosting. In order to increase host managerial skills, Airbnb can develop on-line training programs to enable the development of particular hosting skills (e.g., boundary setting, house rules, online, F2F and cross-cultural communication). Airbnb might provide training around how to better standardize the accommodation experience. Airbnb can also foster online host communities, so hosts can learn from other hosts. Airbnb might seed these online discussions around particular hosting issues, such as setting accommodation rules and boundaries, or dealing with complaints or poor guest behaviors. Airbnb should also reduce their efforts to get hosts to reduce pricing, especially directed at novice hosts, as this can reduce their sense of efficacy with hosting. Hosts also desire a greater degree of support from Airbnb when issues arise. This might include advice on how to deal with conflicts when they occur. Airbnb might also help hosts to set more realistic expectations around the hosting experience, in terms of how much they may earn, the range of guests they can expect, and the level of effort required to host. In addition, recent research shows that travelers are very reluctant to book shared flats on Airbnb during the COVID-19 pandemic but full units are preferred to hotel rooms (Bresciani et al., 2021). Thus, if Airbnb can help hosts to develop rules to assure physical distance (e.g., use of masks, self-check-in), this may help to reduce guest concerns toward shared flat options (Bresciani et al., 2021).

Our research has a number of limitations that should be considered in the interpretation of the findings presented here. First, the present study has a relatively small sample size and the generalizability of the findings to the wider population of Airbnb hosts is unable to be determined. Second, as with any type of qualitative research, the findings cannot be generalized to all Airbnb hosts. Third, host participants in the present study were from a single geographical region in New Zealand and the majority of our hosts were female. Future research may seek to adopt quantitative approaches, such as surveys, to determine the extensiveness of phenomena reported here among a larger more representative sample. Future research would also benefit from examining hosts from different geographic regions, or hosts that use other hosting sites to determine whether hosting on those sites enhance or diminish wellbeing in a similar manner to our findings. Finally, our data was collected before the COVID-19 pandemic, thus future research is necessary to determine how are findings are impacted by host concerns about the pandemic.

## Data Availability Statement

The raw data supporting the conclusions of this article will be made available by the authors, without undue reservation.

## Ethics Statement

The studies involving human participants were reviewed and approved by the University of Canterbury Human Ethics Committee. The patients/participants provided their written informed consent to participate in this study.

## Author Contributions

Both authors contributed equally to this research and approved it for publication.

## Conflict of Interest

The authors declare that the research was conducted in the absence of any commercial or financial relationships that could be construed as a potential conflict of interest.

## Publisher’s Note

All claims expressed in this article are solely those of the authors and do not necessarily represent those of their affiliated organizations, or those of the publisher, the editors and the reviewers. Any product that may be evaluated in this article, or claim that may be made by its manufacturer, is not guaranteed or endorsed by the publisher.

## Appendix

### Interview Questions

1. How long have you been offering Airbnb accommodation?
2. Do you have other experience in the accommodation sector? Can you describe those?
3. How often do you get bookings through the Airbnb website?
4. Do you list your property on other online sites (e.g., bookabach, booking.com)?

- What is different/better/worse about hosting through AirBnB?

5. What is the nature of the accommodation you are offering?
6. Do the guests have exclusive use of the property during their stay or is the space shared with you?
7. How much do you interact with your guests during their stay?
8. What do you enjoy about the interactions with your guests?
9. What are the most important benefits you get from participating as a host through AirBnB?
10. Tell me about your best/worst hosting experience?
11. Tell me about what you enjoy/don’t enjoy about hosting guests through Airbnb?
12. Do you have any security concerns when hosting guests?
13. Have you found any unexpected obstacles in your experience hosting through Airbnb? If so, can you describe those obstacles?
14. Are there any other things you would like to share with me about your AirBnB hosting experience?
15. What else should I have asked you about your Airbnb hosting experience?
